# Seroprevalence of Hydatidosis in People Referring to Reference Laboratory of Gorgan, Golestan Province, Northern Iran 2017

**Published:** 2019

**Authors:** Saman FATHI, Reza GHASEMIKHAH, Rasool MOHAMMADI, Farideh TOHIDI, Mitra SHARBATKHORI

**Affiliations:** 1. Student Research Committee, Arak University of Medical Sciences, Arak, Iran; 2. Infectious Diseases Research Center (IDRC), Arak University of Medical Sciences, Arak, Iran; 3. Department of Parasitology and Mycology, School of Medicine, Arak University of Medical Sciences, Arak, Iran; 4. Department of Epidemiology and Biostatistics, School of Public Health and Nutrition, Lorestan University of Medical Sciences, Khorramabad, Iran; 5. Laboratory Sciences Research Center, Golestan University of Medical Sciences, Gorgan, Iran; 6. Infectious Diseases Research Center, Golestan University of Medical Sciences, Gorgan, Iran; 7. Department of Parasitology and Mycology, School of Medicine, Golestan University of Medical Sciences, Gorgan, Iran

**Keywords:** Seroprevalence, Human hydatidosis, ELISA, Iran

## Abstract

**Background:**

Hydatidosis is a neglected global zoonotic disease, caused by larval stage of the cestode *Echinococcus granulosus* in human and animal. Because of high economic and medical importance of the disease, this study was performed to find the seroprevalence of human hydatidosis in Gorgan City, Golestan Province, northern Iran.

**Methods:**

In this cross-sectional study, blood samples were collected from people referring to Reference laboratory of Golestan University of Medical sciences in 2017. A relevant questionnaire was completed for demographic data for each person. *Echinococcus* IgG antibody was investigated by ELISA using native antigen B. The data were analyzed using SPSS software applying logistic regression.

**Results:**

Overall, 612 blood samples were collected. Cut-off was considered 0.29. Sixteen cases (2.6%) were seropositive for hydatidosis. The seroprevalence of hydatidosis was 2.3% and 4.7% among males and females, respectively. There was no statistically significant correlation between the hydatidosis and investigated variables such as sex, age, tribes, residence, education, etc.

**Conclusion:**

The prevalence of human hydatidosis shows approximately the same range as other regions of Iran. Although due to the neighboring the Mazandaran Province reported as the highest seroprevalence of hydatidosis, we expected more rate of seropositivity.

## Introduction

Hydatidosis or cystic echinococcosis (CE), caused by larval form of the several species belong to the genus *Echinococcus*, is one of the major zoonotic diseases in the world that creates considerable economic losses and public health issues. WHO has included echinococcosis in the list of neglected tropical diseases (1, 2). The most prevalent species of *Echinococcus* that globally infects human is *E. granulosus* which is a complex of species and genotypes containing differences in their life cycle patterns and host range (3). Human infect via ingestion of helminths' egg through different ways such as eating vegetables contaminated to stool off canid hosts, infected by eating the viscera of domestic or wild livestock involving hydatid cysts (4, 5).

The disease is common in many sheep farming regions like Australia, New Zealand, Asia, eastern and southern Europe, South America, Mediterranean coasts and Middle East including Iran (4, 6). Echinococcosis is maintained in three distinct cycles in Iran, a domestic dogs/livestock cycle, a desert dogs/camels cycle and a wild cycle including wild carnivores/wild ruminants (7).

Iran has been announced as an endemic country for echinococcosis by WHO and different researches confirmed this issue (8, 9). The number of asymptomatic people living in Iran was estimated 635,232, and total annual cost of cystic echinococcosis is US$ 232.3 million (10). The country has proper situation for infecting with hydatidosis including high rate of dogs infected with *E. granulosus*, humidity in northern Provinces, food habit of using raw vegetable, carrot juice, etc. (11). So far, many studies have been performed in different regions of Iran to find the seroprevalence of human hydatidosis (8). The prevalence rate has been reported from 1.2% to 31.6% in different Provinces (7, 12). The asymptomatic duration is too long and the disease might be recognized even more than 20–25 years after infection (7). Human hydatid diagnosis based on clinical symptoms is a problem and requires multiple paraclinical, serological investigations and imaging techniques (Xray, CT, MRI, etc.) to confirm the clinical suspicion. Many immunodiagnostic tests have been established to detect hydatidosis, but mostly ELISA test using native antigen B served as a valuable and efficient test to detect specific antibodies and the seroprevalence of the diseases (8, 13, 14).

Considering the high medical and economic importance of the disease, this study aimed to determine the prevalence of human hydatidosis using ELISA method in people referring to Reference laboratory of Gorgan, Golestan Province, northern Iran 2017.

## Materials and Methods

### Samples

**Fig. 1:**
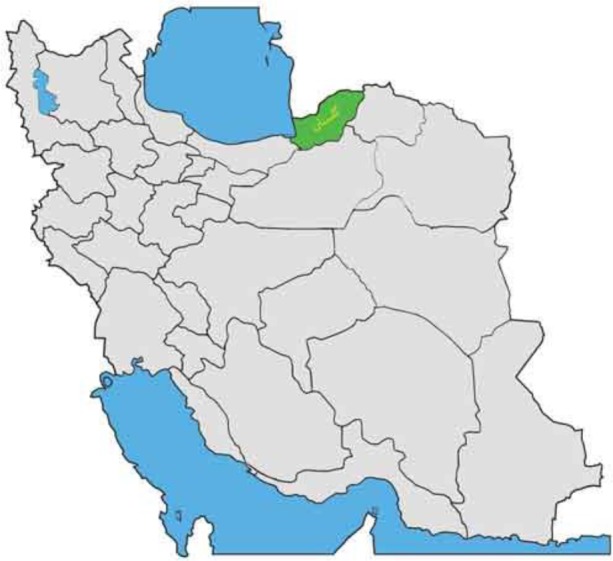
Location of Golestan Province in Iran, showed in green color

This descriptive cross-sectional study was conducted from Feb to Jun 2017 in Gorgan City, capital of Golestan Province, Southeastern the Caspian Sea, north of Iran (Fig. 1).

Gorgan has a population around 300000 people. The city has a humid climate. In the current survey, samples size was calculated considering prevalence of 2.15% (15), a degree of precision of 4 (d=0.04) and 95% confidence interval. Consequently, the sample size was obtained by about 600 people.

The study was approved by Ethics Committee of the Golestan University of Medical Sciences with confirmation No. 1394.IR.GOUMS.REC.307. Samples were collected from randomly people attending to Reference laboratory of Golestan University of Medical sciences. Individual informed written consent was taken from subjects prior to registering in the study.

A detailed demographic data and relevant history were recorded in questionnaires such as gender, age, living area (urban/rural), tribes (Fars, Sistani, Turkmen, …), literacy, occupation, habit of chewing nails, history of soil or dogs contact, eating raw vegetables, etc. Three milliliter of venous blood sample was taken from each study subject and sera were separated by centrifugation at 3000 rpm for 5 min and stored at −20 °C. The sera were transferred in cold conditions to the parasitology laboratory of Tehran University of Medical Sciences for further analysis.

### Antigen

Crude hydatid cyst fluid (HCF Ag) was aspirated from hydatid cysts taken from infected livers or lungs of sheep at the local slaughterhouses of Tehran. Antigen B was purified and extracted (16).

### ELISA test

Examining *Echinococcus* IgG in the samples was conducted in 96 well microplates (Nunc, Denmark) using ELISA. IgG-ELISA test was performed on serum samples (17). The optical density (OD) at 492 nm was measured using an ELISA plate reader (State Fax® 2100, Awareness, USA). Totally, 30 sera from healthy volunteers had been collected during the previous studies were tested to find the cut-off. The cut-off point was assigned as 3SD above the mean of controls (17).

### Data statistical analysis

In order to examine the relationship between each variable and risk of human cystic echinococcosis, odds ratio (OR) and 95% confidence interval (CI) were estimated using logistic regression. *P*less than 0.05 was considered significant. The data were analyzed using SPSS software ver. 22 (Chicago, IL, USA).

## Results

**Fig. 2:**
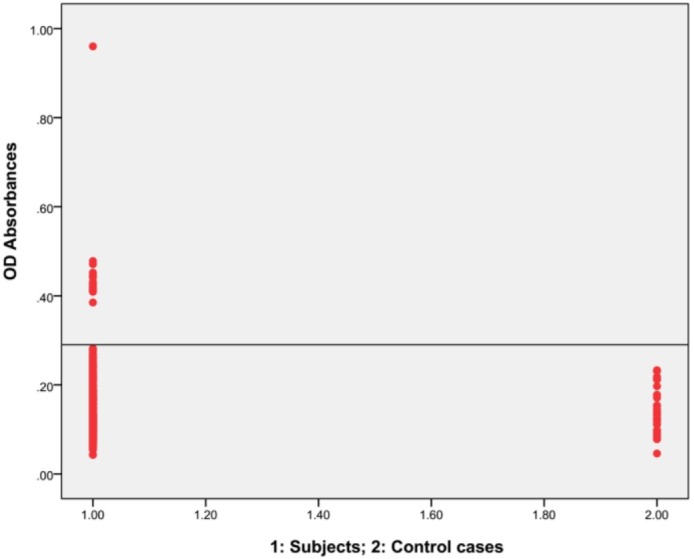
Analysis of sera from people referring to Reference laboratory of Gorgan, Golestan Province, northern Iran and normal controls by IgG-ELISA employing antigen B. Cut off= 0.29. Serum samples obtained from subjects (612, Lanes 1), and normal controls (30, Lanes 2)

Totally 612 blood samples were collected. Eighty-six (14.1%) and 526 (85.9%) were male and female, respectively. Cut-off was obtained as 0.29 and each OD absorbance higher than this rate was considered as positive (Fig. 2). Sixteen (2.6%) people including 4 males and 12 females were positive for hydatidosis (Fig. 2). The seroprevalence of hydatid cyst was 2.3% and 4.7% among males and females, respectively, which showed no significant difference. The infection rate was 1.4% and 3% in age group below and above 40 yr old, respectively. There was not any significant relationship between the age and the disease. Distribution of the tribes were as follows, Fars (72.2%), Sistani (21.7%), Torkmen (2%) and 4.1% belonged to other different tribes. The infection rate in Fars tribe (2.5%) was lower than total of other tribes (2.9%) with showed no significant difference. There was no significant relationship between the tribes and the disease. Overall, 409 people were urban (66.8 %) and 203 were rural (33.2 %). Data analysis did not show any significant difference regarding the residency place. 2.4% and 3.9% of educated and non-educated people were infected with hydatidosis, respectively. Analysis showed no significant correlation between the disease and level of literacy.

Regarding occupation, the highest and lowest rate of infection were found among farmers (3.8%) and housekeepers (2.1%), respectively and no statistically significant differences were seen among different jobs. The seropositive rate in people with history of chewing nails was lower (2%) than others (2.7%) with no significant difference. The infection rate was higher in people with history of contact with soil (4% vs. 2.1%), dog (4.3% vs. 2.5%) and consuming raw vegetables (2.8% vs. 1.9%), which showed no significant difference (Table 1).

**Table 1:** Logistic regression analysis of seropositive cases of hydatidosis according to sex, age, tribes, residence, occupation, education, habit of chewing nails, contact with soil and dogs, consuming raw vegetables

***Variables***		***Seroprevalence***			
		***No. of Examined (%)***	***No. of Positive (%)***	***Odds Ratio (95% CI^a^)***	***P***
Sex	Female	526 (85.9)	12 (2.3)	Reference^b^	0.211
	Male	86(14.1)	4.0 (4.7)	2.089 (0.658, 6.634)	
Age (yr)	≤ 40	148 (24.2)	2.0 (1.4)	Reference	0.282
	> 40	464(75.8)	14 (3.0)	2.271 (0.510, 10.11)	
Tribes	Fars	442 (72.2)	11 (2.5)	Reference	0.754
	Non-Fars	170 (27.8)	5.0 (2.9)	1.187 (0.406, 3.47)	
Residence	Urban	409 (66.8)	12 (2.9)	Reference	0.485
	Rural	203 (33.2)	4.0 (2.0)	0.665 (0.212, 2.09)	
Occupation	Housekeeper	425 (69.4)	9(2.1)	Reference	
	Farmer	26 (4.2)	1(3.8)	1.849 (0.225, 15.175)	0.567
	Other	161 (26.3)	6(3.7)	1.789 (0.627, 5.110)	0.277
Education	Educated^c^	510 (83.3)	12 (2.4)	Reference	0.370
	Non-educated	102 (16.7)	4.0 (3.9)	1.694 (0.535, 5.361)	
Habit of chewing nails	No	562 (91.8)	15 (2.7)	Reference	0.777
	Yes	50 (8.2)	1.0 (2.0)	0.744 (0.096, 5.75)	
Contact with soil	No	438(71.6)	9.0 (2.1)	Reference	0.177
	Yes	174 (28.4)	7.0 (4.0)	1.998 (0.732, 5.452)	
Contact with dog	No	566 (92.5)	14 (2.5)	Reference	0.450
	Yes	46 (7.5)	2.0 (4.3)	1.792 (0.395, 8.14)	
Consuming raw vegetables	No	106 (17.3)	2.0 (1.9)	Reference	0.608
	Yes	506 (82.7)	14 (2.8)	1.480 (0.331, 6.609)	

a CI: confidence interval

b Reference: The level of variable which other levels compared with that.

c Educated: defines as primary, secondary school, high school and university

## Discussion

In the present study, a seroprevalence of 2.6% (of 612 cases) was detected for human hydatidosis in people referring to Reference laboratory of Gorgan, using ELISA test.

Gorgan city has a humid climate that creates a proper condition for *Echinococcus* eggs to stay alive and have more chances of transmission to intermediate hosts. Due to wet weather conditions, high rate of livestock raising and grazing, existence of stray dogs as an important definitive host, around the Gorgan, the cycle of transmission of hydatidosis is easily possible. In this study, we challenged the seroprevalence of human hydatidosis in Gorgan city, northern Iran to complete the prevalence puzzle of the disease in the country.

Human and animal hydatidosis is endemic in different parts of Iran. Dogs as definitive host have a significant role in transmission of the disease. A prevalence of 5%–49% has been reported for hydatidosis in dogs in different parts of the country (7, 9). The different climate conditions of the country have a significant role on prevalence rate of the disease. Recently in Mazandaran, a northern Province, 28.6% of 42 dogs 18.7% of 16 jackals were infected to *E. granulosus* (18).

The infection rate of *E. granulosus* in various domestic livestock has been stated to be 24.41%, 8.51%, 18.89%, 35.76%, and 35.21% in sheep, goat, cattle, buffalo, and camels, respectively (8). Annual incidence of human CE globally is from 1 to 200 per 100,000 residents (4). HCE is responsible for almost 1% of admission to surgical wards in Iran and the annual incidence varies from 0.6 to 3 per 100,000 populations in different regions of the country (7).

To date, many studies based on different serological procedures have been performed to find the rate of human echinococcosis in different regions of Iran. Using various serological method might be a reason for different results. We used ELISA method with antigen B because this test is acceptable, efficient, easy and affordable with high sensitivity and specificity (8). Recently, in Mazandaran Province, southern Caspian Sea, a seroprevalence of 31.6% for hydatidosis reported that is the highest seroprevalence have ever been recorded in the country also in the world (12).

The high infection rates of the hydatidosis have been recorded from western and southwestern of the country. For example, 15.4% in Khorramabad (19), 13.78% in Khuzestan Province, 13.7% in Fars Province (20). Lower prevalences have been reported from rural communities of Kerman 7.3% (21), Charmahal and Bakhtiari 4.8% (22), Arak 3.46% (23), Sarab 3.2% (24), nomads tribes from southwest of Iran 2.8% (25), Rafsanjan 1.83% (26), Qom 1.6% (27), Ardabil 1.79% (28), Tehran 1.63% (29), Isfahan and suburb 1.1% (17). Differences in seroprevalences originate from differences in climates, culture and habit of people in various areas (17).

In Golestan Province, a seroprevalence of 2.15% was reported using ELISA method (15), that is in concordance with our study (2.6%).

In this study, the infection rate in women (4.7%) was more than men (2.3%). That is in agreement with previous study in Golestan Province (15) and other studies which reported higher infection rates in women compare to men (22, 23, 30–32). This could be due to more involvement of women in gardening or cooking activities such as cleaning vegetables etc. Culture and environment of each region is an important factor in prevalence of hydatidosis. Since, where men have more involvement in farming and ranching activities leading to continuous contact with stool of dogs, studies have reported the higher infection rate in men than women (12, 17, 19, 25, 33, 34). In our survey, similar to previous study in Golestan Province (15) there was no significant difference observed between the hydatid cyst and gender.

Hydatidosis can involve people of any ages, from less than one to over seventy-five years old, and generally increases with age (32). In the present study, seroprevalence of hydatidosis was not statistically significant regarding the age group. Our data showed that people above 40 yr old have more chance of seropositivity of the disease than younger age group with no significant differences. That is almost agreement with another study in Golestan Province which reported the highest infection rate in age group of 40–49 yr old (15). Other studies indicated different results. The highest number of hydatidosis cases were reported in age groups: 10–19 yr in Zanjan (35), below 15 yr in India (34), 20–29 yr in Khorramabad (19), 20–39 yr in Kerman (21), 30–39 yr in Turkey (32), 40–49 yr in Arak (23), above 50 yr in Behbahan (25), 60–69 yr in Isfahan (17, 26), 60–90 yr in Ardabil (28), above 60 yr in Greece (34). Because of long prepatent period in hydatidosis it is dificult to precise detection of most infected age group in regarding the disease (4).

This study did not indicate any significant difference between different tribes and hydatidosis. The seropositivity in non-Fars tribes was more than Fars tribe (2.9% vs. 2.5%).

Regarding to residence, urban life (2.9%) indicated no significant difference with rural life (2%). That is in concordance with study of Baharsefat and colleagues that reported 2.47% Vs. 2.45% prevalence for urban and rural life in Golestan Province, respectively (15). However, most previous studies have reported a higher risk of hydatidosis for rural life (12, 19, 26, 33, 36).

Habit of chewing nails showed no significant difference with seropositivity in our study.

This study showed a higher seropositivity in subjects having contact with soil (4% vs. 2.1%) and dogs (4.3% vs. 2.5%) which is in concordance with other studies (12, 20, 30, 37). However, the differences were not significant in our study.

Non-educated people were more infected (3.9%) than educated ones (2.4%) to hydatidosis. That is in agreement with some previous studies (15, 19, 23, 26, 28, 32) and disagreement with study that surprisingly educated people were 1.5 times more infected to hydatidosis than non-educated (12). Literacy indicated no significant difference in our study.

People consuming raw vegetables were more seropositive (2.8% vs. 1.9%), that showed no significant difference with the disease. Eating raw vegetables have been reported as a significant factor for seropositivity in the Mazandaran Province, neighboring the Golestan Province.

Overall, due to relatively high prevalence (2.6%) of hydatidosis among people referring to the Reference laboratory of Gorgan, future more extensive studies in the city and the province is suggested and control strategies such as preventive measures and public health education should be considered by authorities.

## Conclusion

The prevalence of human hydatidosis in this study shows almost the same range as other regions in Iran. However, this study performed only on the people referring to the Reference laboratory of Gorgan and cannot be generalized to entire the city. Thus, due to importance of the disease, and neighboring the Mazandaran Province with the highest prevalence of hydatidosis, finding the seroprevalence of hydatidosis in a comprehensive study in Golestan Province should be considered in future researches.
